# Integrative Neuromuscular Training in Adolescents and Children Treated for Cancer (INTERACT): Study Protocol for a Multicenter, Two-Arm Parallel-Group Randomized Controlled Superiority Trial

**DOI:** 10.3389/fped.2022.833850

**Published:** 2022-03-14

**Authors:** Peter Schmidt-Andersen, Martin Kaj Fridh, Klaus Gottlob Müller, Anna Pouplier, Lisa Lyngsie Hjalgrim, Avery D. Faigenbaum, Kjeld Schmiegelow, Henrik Hasle, Sine Lykkedegn, He Zhang, Jan Christensen, Hanne Bækgaard Larsen

**Affiliations:** ^1^Department of Pediatrics and Adolescent Medicine, The Juliane Marie Center, Copenhagen University Hospital, Copenhagen, Denmark; ^2^Department of Occupational and Physiotherapy, Center of Head and Orthopedics, Copenhagen University Hospital, Copenhagen, Denmark; ^3^Department of Clinical Medicine, Faculty of Health and Medical Sciences, University of Copenhagen, Copenhagen, Denmark; ^4^Department of Health and Exercise Science, The College of New Jersey, Ewing, NJ, United States; ^5^Department of Pediatrics and Adolescent Medicine, Aarhus University Hospital, Aarhus, Denmark; ^6^Department of Pediatric Hematology and Oncology, H.C. Andersen Children's Hospital, Odense University Hospital, Odense, Denmark

**Keywords:** childhood cancer, integrative neuromuscular training, rehabilitation, during treatment, survivorship, muscle strength, metabolic syndrome

## Abstract

**Background:**

Improved survival rates for children and adolescents diagnosed with cancer call for novel strategies for reducing short- and long-term treatment-related side effects. These include the physical and metabolic sequelae that are exacerbated by sedentary behavior and treatment-induced toxicities. We aim to investigate the effect of an integrative neuromuscular training intervention during the first 6 months of anti-cancer treatment primarily on muscle strength, and secondarily on exercise capacity, physical function, markers of metabolic syndrome, dysmetabolism, and health-related quality of life during and after ended treatment.

**Methods:**

One hundred and twenty-seven children and adolescents, newly diagnosed with malignant and benign neoplasia, aged 6–17 years, and treated with chemotherapy or radiation will be randomized to either the intervention or the control arm of the study. The intervention group will, in addition to usual care, be offered a combination of 6 months of supervised physical exercise (integrative neuromuscular training) and home-based exercise. The active control group will, in addition to usual care, receive information along an unsupervised written home-based training program. All participants, including parents, will receive information about the importance of physical exercise during the course of cancer treatment, at the start of treatment, and in 5 monthly sessions. The primary outcome is measured in terms of isometric quadriceps muscle strength. Secondary outcomes include muscle strength and endurance, markers of metabolic syndrome and dysmetabolism, exercise capacity, physical function and activity, days of hospitalization, and health-related quality of life. Assessment will be conducted at treatment initiation (baseline), at 3 and 6 months after inclusion, and 1 month and 1 year after ended treatment. The primary endpoint for lower-body muscle strength is at 6 months after treatment initiation. The effects of the intervention will be evaluated through a constrained linear mixed model.

**Discussion:**

This national randomized controlled study has the potential to provide new knowledge concerning the short- and long-term effects of a novel, inclusive approach for youth exercise programming (integrative neuromuscular exercise) in children and adolescents during anti-cancer treatment. Using a pragmatic, low-cost, and time-efficient training design, this intervention can be easily adapted to both hospital and home settings.

**Clinical Trial Registration:**

ClinicalTrials.gov (NCT04706676), first released January 5, 2021.

## Introduction

In the Western world, the 5-year survival rate for children and adolescents diagnosed with cancer has improved progressively over the last 3 decades; from 72 in 1985 to above 85% in 2017 (1, 2). However, this improved rate is accompanied by an increase in both short- and long-term side effects (1, 2) and calls for novel strategies that work beyond sole survival as most children enter a negative loop where treatment-induced toxicities and a sedentary lifestyle exacerbate the physical deficits of cancer treatment (3–14).

Children and adolescents diagnosed with cancer are predominately treated with chemotherapy, radiotherapy, glucocorticoids, and surgery causing well-documented side effects, including damage to skeletal muscles, the central and peripheral nervous systems, and impaired cardiorespiratory fitness. This results in impaired gait (walking distance and reaction time) and balance, and it leads to fatigue and reduced physical activity (10, 15–18). Collectively, these factors have significant negative implications for physical health outcomes, including risk of muscle atrophy (10, 14, 19, 20), which persist into adulthood, as approximately two-thirds of cancer survivors have shown to have at least one chronic health condition 30 years after treatment initiation (8).

Skeletal muscles serve fundamental functions, ranging from generating mechanical force and mobility to regulating whole-body metabolic homeostasis (15). Hence, muscle atrophy and altered body composition with lower lean body mass and skeletal muscle index, seen after cancer treatment, threaten independent living due to reduced physical ability (15, 21–23). Muscle atrophy may also play an essential role in the development of dysmetabolism in long-term survivors, i.e., studies have reported increased prevalence of metabolic syndrome with physical inactivity being a predominant risk factor (OR, 1.7; 95% CI, 1.1–2.6) (24).

These severe physical and metabolic disturbances may be founded early during cancer treatment, as lower-body muscle strength and cardiorespiratory fitness are significantly decreased by 21 and 42%, respectively, within the first 30 days of treatment in children and adolescents compared to age- and sex-matched controls (10, 18), which highlights the need for early exercise interventions (13).

Previous studies in pediatric oncology patients indicate that exercise interventions are generally safe, feasible, and have beneficial preserving effects on muscle strength, cardiorespiratory fitness, and physical functioning during cancer treatment (18, 25–28). Furthermore, children are interested and can be motivated to engage in exercise and physical activity while hospitalized despite cancer disease and intensive chemotherapy (13, 18, 29).

In general, the body of evidence concerning the effectiveness of exercise interventions during anti-cancer treatment in children is based on studies with small sample sizes, heterogeneous aims, interventions, and outcomes; using either broadly defined or undefined exercise interventions with a low grade of reproducibility (9, 11, 13, 30–52).

An emerging, more inclusive, concept of exercise is integrative neuromuscular training; a conjunction of different types of physical exercise with potential neuromuscular output designed to enhance both health- and skill-related components of physical function (53). Moreover, it is time-efficient, can be adapted to both hospital and home settings, and is developmentally appropriate for both children and adolescents. Accordingly, this type of exercise is thought to counteract both lifestyle and potentially treatment-induced neuromuscular deficits and improve physical function, such as walking, running, lifting, and balance; fundamental movement skills for achieving a long-term physically active and healthy lifestyle (15, 32, 34, 37, 54, 55).

Quasi-experimental and controlled studies have underscored how 7–12 weeks of integrative neuromuscular training can improve muscular strength, fundamental movement skills, and selected measures of physical fitness compared to physical education classes and customary sports in healthy children and adolescents (5–14 years) (56–60).

Although no studies have been conducted on children and adolescents during prolonged periods of hospitalization nor during cancer treatment, integrative neuromuscular training appears as a feasible exercise modality due to its age- and skill-appropriate approach to progressive exercise targeting neuromuscular deficits. Furthermore, its challenging, motivational, play-and-game approach to exercise can potentially improve adherence and long-term lifestyle behavior in children surviving cancer.

The study is based on the overarching hypothesis that supervised structured integrative neuromuscular training initiated at the time of diagnosis effectively prevents deficits in muscle strength 6 months after initiated treatment.

The primary objective of this study is to investigate the effects of a 6-month integrative neuromuscular training intervention compared with unsupervised home-based exercise on isometric knee extension strength in children and adolescents (6–18 years) during anti-cancer treatment. Our secondary objectives are to investigate the effects of the intervention on markers of metabolic syndrome, days of hospitalization, health-related quality of life, upper-body muscle strength, exercise capacity, physical function, physical activity behavior, and body composition.

## Methods and Analysis

This protocol is reported according to the Standard Protocol Items: Recommendations for Interventional Trials (SPIRIT) (61).

### Trial Design

The INTERACT study is a national multicenter, two-arm parallel-group, randomized controlled superiority trial based on empirical evidence within the research group (13, 18, 43, 62) and methodical recommendations from current evidence (27). The primary endpoint is at 6 months after inclusion, and follow-up will be 12 months after ended treatment.

### Setting

The three of four centers for pediatric oncology in Denmark will functions as trial sites: Copenhagen University Hospital, Rigshospitalet; Aarhus University Hospital; and Odense University Hospital.

### Eligibility Criteria

Children and adolescents with newly diagnosed malignant and benign neoplasia aged 6–17.9 years and admitted from January 2021 for treatment at the departments for pediatric oncology will be eligible for inclusion. Diagnoses include malignant and benign neoplasia treated with chemotherapy and/or irradiation. Children with a severe mental and/or physical disability (i.e., participants where all types of physical training and testing of physical function are contraindicated), terminal illness, and individuals unable to communicate in Danish will be excluded.

When answering patient-reported outcomes, the parent(s) will be used as informants to answer proxy questionnaires and provide sociodemographic data on behalf of their child.

### Recruitment

All eligible participants and their parents will receive the information about the study within 2 weeks of treatment initiation by the treating physician at the clinical ward. If interested, a member of the research team, a project nurse or physiotherapist, will provide oral and written information about the study to the child and parents, in a quiet and undisturbed environment on the ward.

Participants who are willing to participate will sign the informed consent before any study-related procedures are initiated. When informed consent for participation is obtained, the recruitment staff will schedule the baseline assessments in the local occupational- and physiotherapy department, which will be conducted before randomization.

### Integrative Neuromuscular Training

In addition to usual care, the intervention group will receive integrative neuromuscular training (INT) for 6 months. An overview of the components of the intervention (and active control group) can be found in Table 1.

**Table 1:** Overview of content in the intervention and active control group.

	**Study interventions**	
	**(Experimental) integrative neuromuscular training**	**(Comparator) active control group**
**Description**	Supervised neuromuscular exercise during admissions and visits to the outpatient clinic, containing elements of strength, motor skill, dynamic stability, core-focused strength, and agility exercises (prescribed according to age and training experience) Home-based exercise during weeks without visits to the hospital	Unsupervised home-based training program consisting of combined aerobic, strength, and stretching exercises (described in Additional file 1)
**Duration**	6 months of exercise initiated 2 weeks within start of cancer treatment	
**Recommended frequency (minimum session/weekly)**	2 training sessions/week for the first 7 weeks 3 sessions/week from weeks 8–24	2 sessions/week
**Recommend time/session**	15–35 min	15–20 min
**Recommended no. of exercises**	2–6	3
**Usual care**	Both groups will receive usual standardized hospital care, including physiotherapy if needed.	
**Motivational counseling**	Each child and their parents will participate in a monthly 15–30-min motivational counseling session.	

*Description of content in the intervention (experimental) and active control (comparator) arms of the study*.

All participants are encouraged to participate in a minimum of two training sessions per week for the first 7 weeks and three sessions per week in weeks 8–24. Usually, during the first 6 months of treatment, all participants indifferent of cancer type will either be hospitalized or have outpatient appointments every week. Hence, at least one supervised training will be planned every week. All remaining training sessions will therefore be conducted as either supervised or home-based training, depending on admission. If there are weeks without any hospital or outpatient clinic visits, the training sessions will all be conducted as home-based training, and the participants will receive a phone call or text message from the intervention physiotherapist concerning questions, exercise choice, and exercise intensity.

Based on individual needs and where applicable, parents will be instructed to conduct INT at home. When relevant participants will be provided with exercise equipment corresponding to the child's age and fitness level (e.g., fitness ropes, medicine ball, dumbbells).

Integrative neuromuscular training contains a range of developmentally appropriate activities that target general and specific strength and conditioning elements, such as strength, power, motor skills, dynamic stability, core-focused strength, and agility (53, 63). INT can be camouflaged as games and play or performed as a structured strength and conditioning program, depending on the participant's age, motor skill level, and daily variations in side-effects (nausea, fatigue, dizziness, pain). Unlike more traditional types of physical activity (e.g., walking, cycling), integrative neuromuscular training targets neuromuscular deficits by stimulating neural plasticity, alerting motor unit recruitment, firing frequency, and synchronization of motor unit activation (15, 32, 34, 37, 54, 55). The intervention is designed to enhance both health- and skill-related components of physical fitness.

To increase adherence, training intensities (load or level of difficulty) and length of training sessions (training volume and rest periods) will be periodized according to the participants' chemotherapy cycles, where applicable, to accommodate potential side effects, primarily treatment-related fatigue (64). An example of a training plan adjusted to a low-risk treatment protocol for acute lymphoblastic leukemia (ALLtogether 2018; ClinicalTrials.gov NCT04307576) can be seen in Supplementary Figure 2.

Training intensity (load or level of difficulty) and length of training sessions are adjusted throughout the treatment trajectory and expected to be considerably lower the first week following chemotherapy. The purpose of this pre-emptively reduced intensity and volume is to (1) encourage participants to attend exercise, even though physical symptom burden may be more extensive during these periods and (2) prescribe manageable exercise accommodating the symptom burden (64). Furthermore, to familiarize the participants with physical exercise, in a period of transition from everyday life to life with cancer, including treatment regimens and hospitalization, the initial weekly training frequency will be fixed to a minimum of two training sessions per week for the first 7 weeks, and a minimum of three sessions per week in weeks 8–24.

### Health Counseling/Motivational Intervention

Due to the strain related to the anti-cancer treatment, motivation is paramount in this setting. Each child and their parents in both groups will participate in a monthly 20-min health counseling session to adjust the intervention according to the child's needs and preferences.

The sessions are based on self-determination theory (65), describing the interplay between external and internal motivation forces, defined within three innate psychological needs/parameters: autonomy, competence, and relatedness. Practically, these sessions will follow a semi-structured interview protocol involving: (1) autonomy: Each participant has the option to change the training program according to their needs, skill level, and presence of symptoms using cooperative planning (co-creation); (2) competence: It must be apparent for the participants that the training sessions maintain or develop their physical function by tracking progress in the exercise diaries (e.g., number of repeated exercises, loads, difficulty of exercise). Furthermore, if applicable, the participant sets a monthly goal for participation level within the international classification of functioning, disability, and health (ICF) (66); (3) relatedness is achieved by putting the potential effects of exercise into a social context; e.g., that through exercise, they can partake more easily in social relations on equal terms with peers.

The goal is to achieve internal motivation to engage in exercise and physical activities; that is, to design the exercise program so that the child engages in the exercises for the fun of it.

### Home-Based Training Program

The active control group will receive a home-based training program consisting of strength and stretching exercises for lower and upper body (see Supplementary Figure 1). The participants can choose from two or three stretching, lower- and upper-body resistance exercises, respectively, and they are asked to perform three sets of 10 repetitions for each resistance exercise. All exercises use body weight as resistance but can be progressed in terms of level of difficulty. The use of the home-based training program will be monitored with an exercise diary.

### Usual Care

Both groups will receive usual standardized hospital care, including physiotherapy, as needed. However, procedures for referrals to physiotherapy and staff resources are different in each center. At Copenhagen University Hospital, Rigshospitalet, children and adolescents are referred to physiotherapy when/if a physical deficit occurs (e.g., impaired gait and balance, drop feet, surgical operations). In contrast, at Aarhus and Odense University Hospital, all children are referred to physiotherapy at diagnosis. At all three centers, physiotherapy resources will be distributed according to the severity of illness and physical deficits.

### Randomization

Following baseline assessment, participants will randomly be assigned to either the intervention group (integrative neuromuscular training + motivational-counseling sessions + usual care) or active control group (home-based training program + motivational-counseling sessions + usual care) by a blinded statistician using a computer-generated concealed allocation procedure to secure a proportionate stratified random sample with a (2:2) allocation. Participants will be stratified by sex, pubertal stage, and diagnosis as treatment for (1) solid tumors, (2) CNS-tumors, and (3) treatment for hematologic malignancy.

Baseline assessors and the statistician will be blinded to the allocation of participants; however, due to the nature of the intervention, neither participants nor intervention staff will be blinded throughout the intervention.

### Fidelity

This research project is based on an international collaboration between specialists in metabolism, exercise, and physical activity in pediatric cancer patients. Further, it is based on several years of experience with exercising children and adolescents with cancer through the RESPECT project (REhabilitation including Social and Physical activity and Education in Children and Teenagers with cancer), based at Copenhagen University Hospital, Rigshospitalet. The RESPECT project has shown how children can and will perform safe, in-hospital exercise and how this counteracts side effects resulting from cancer treatment, including loss of fitness and muscle strength, compared with children in pediatric wards in other Danish hospitals (13, 18, 29, 43). Two key principles of RESPECT are early rehabilitation from treatment initiation and supervised exercise, hypothesizing that: (1) Maintaining children's physical function and fitness is easier during treatment than recovering deficits and developing new relationships post-treatment and (2) supervised exercise is more effective than unsupervised exercise.

These two principles will be continued in the INTERACT project. Moreover, the intervention will be evolved to a more structured design, as results from RESPECT suggest, and it will be able to explore potential effects because of its randomized controlled design.

To secure an aligned intervention and reliability of assessment within the three centers, a mandatory two-day workshop (2 × 4 h) is held at each site for the physiotherapist conducting the intervention. The workshop includes a practical introduction to the integrative neuromuscular training intervention, including pro- and regression of exercise intensity or difficulty, and a thorough run-through of all of the physical assessment protocols.

### Outcomes

**Figure 1:**
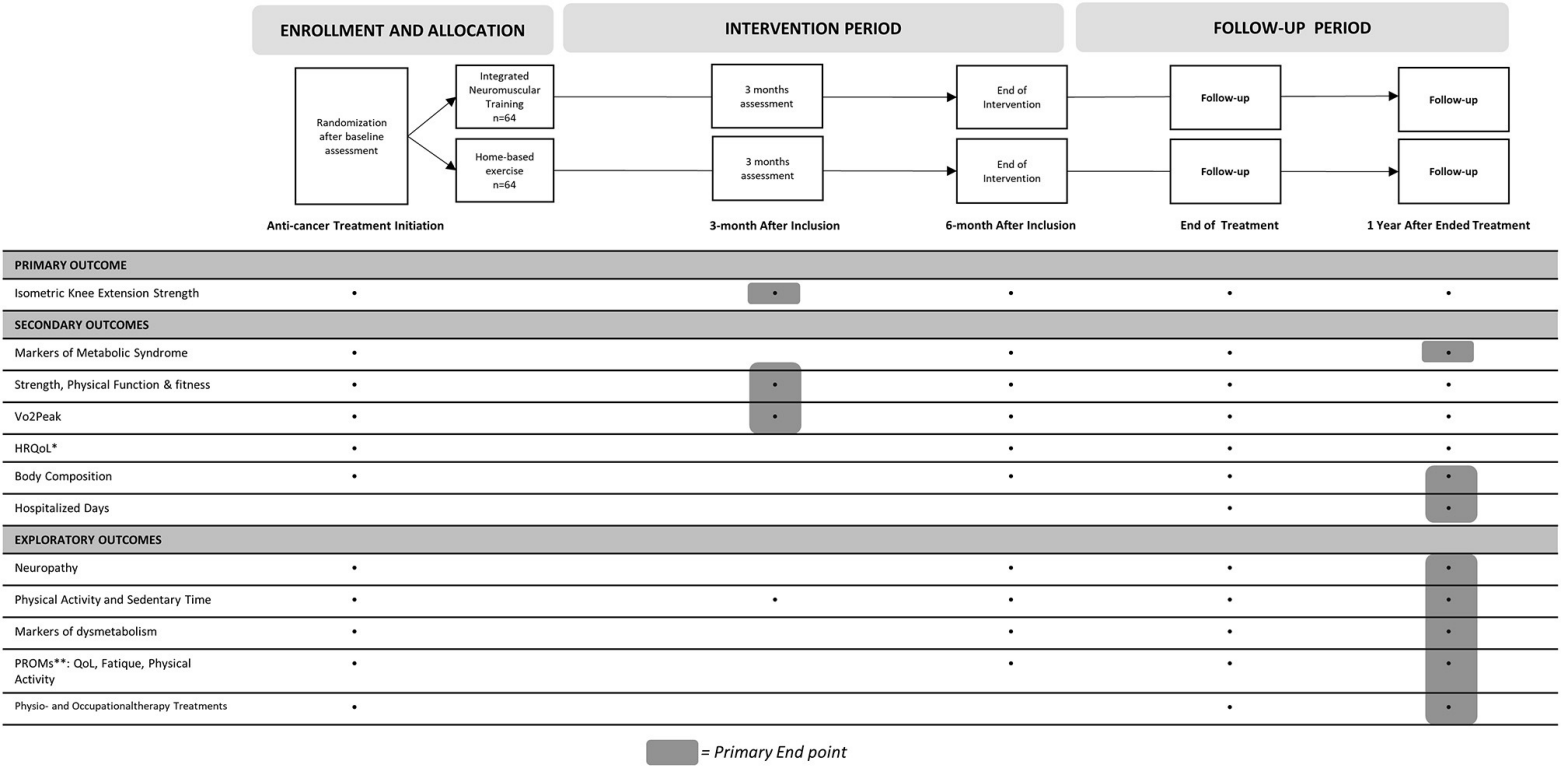
Overview of outcomes and timing of assessment. ^*^, (Health Related) Quality of Life; ^**^, Patient Reported Outcome Measures.

Assessment will be conducted within 14 days after treatment (chemotherapy and/or irradiation) initiation (baseline), at 3 and 6 months after inclusion, and at 1 month and 1 year after ended treatment. An overview of the overall study trajectory, outcomes, and assessment timing is presented in Figure 1.

A complete list of outcomes can be found at ClinicalTrials.gov.

#### Primary Outcome

##### Isometric Knee Extension Strength

Isometric knee extension is tested using a special-build strength ergometer (Gym 2000®, Vikersund, Norway) with a dynamometer (U2A100 kg, Hottinger, Germany) and amplifier. Data is collected using an AD-card (100 HZ) with customized software (LabVIEW ®, National Instruments, Texas, USA). Each participant receives detailed instructions on how to perform each test and is given time to familiarize before each test if needed.

The participant is sitting upright on the bench, with arms hanging alongside the body and hands grasping the bench. Hips and knees are kept in 90 degrees flexion. The height of the bench is adjusted to keep both feet off the ground.

The chain to the dynamometer is adjusted to keep the leg in 90 degrees flexion during muscle contraction. The test is performed unilaterally, primarily on the right leg, unless testing on the right leg is restricted (e.g., due to injury or solid tumors in lower extremity).

The participant is instructed to kick (forward) with maximal force and to keep maximal intensity for at least 5 s. Three attempts with a 2-min break are carried out; however, the participant can try as many attempts as possible if they keep showing improvements. The highest score represents the test score.

#### Primary Secondary Outcome

##### Markers of Metabolic Syndrome (Primary Secondary Outcome)

Metabolic syndrome is based on waist circumference, triglycerides, high-density lipoprotein (HDL) cholesterol, blood pressure, fasting blood sugar, and insulin. Age-based criteria for each parameter concerning metabolic syndrome is defined by the International Diabetes Foundation (67).

Waist circumference is measured in centimeters, after taking several consecutive natural breaths, at a level parallel to the floor, in a midpoint between the top of the iliac crest and the lower margin of the last palpable rib in the midaxillary line following standards described by the World Health Organization (68).

Triglycerides, high-density lipoprotein (HDL) cholesterol, fasting blood sugar, and insulin will be analyzed in blood samples drawn from an antecubital vein or, when possible, through a central or peripheral venous catheter. Samples that have already been collected for routine clinical or research purposes (and stored in an authorized biobank) will also be used in the study to minimize the number of samples taken.

Blood pressure (mmHg) will be measured in the morning using the right arm with the subject sitting.

Although children younger than 10 years cannot be diagnosed with metabolic syndrome, the potential decline or increase in the biological markers (i.e., predisposition for metabolic syndrome) will be investigated in this study.

#### Secondary Outcomes

Secondary outcome measures will include assessment of upper-body muscle strength (measured through isometric bench press; same equipment used for primary outcome), handgrip strength (Jamar, Patterson Medical, Illinois, USA) (69), cardiopulmonary fitness/vo2 peak [through Cardiopulmonary Exercise Test (Cortex, Leipzig, Germany)], walking distance (6-min Walk Test) (70), lower extremity muscle strength and endurance (through 30-s and 1-min Sit-to-Stand Test, respectively) (71, 72), basic functional mobility (through Timed up-and-Go test) (73), body composition [through Whole-Body Dual-Energy X-ray Absorptiometry (DXA) Scan (Lunar, Lunar Corporation Madison, WI, USA)], and quality of life (through PedsQL Generic Core Scale) (74). These outcomes represent direct or surrogate measures of physical fitness, physical function, or quality of life. Each outcome is described in detail at the uploaded protocol at clinicaltrails.gov (NCT04706676).

Further, as a measure of the economic cost of hospitalization, total days of hospitalization will be measured and compared in the two groups after ended treatment.

#### Explorative Outcomes

On an explorative basis, this study will measure neuropathy (through Pediatric Modified Total Neuropathy Score) (75), balance (as Modified Clinical Test of Sensory Interaction in Balance) (76), physical activity and sedentary time through accelerometry (ActiGraph™, ActiGraph LLC, Pensacola FL, USA), muscle power through countermovement jump (FP4, HUR-Labs Oy, Tampere, Finland), markers of dysmetabolism (metabolomics, intestinal microbiota, inflammatory cytokines and mediators, growth and reproductive factors, and macro- and micronutrients collected through plasma, urine and feces samples, and dietary assessment), self- and proxy-reported general physical activity, health-related quality of life (PedsQL 3.0 Cancer Scale) (74), and fatigue (PedsQL Multidimensional Fatigue Scale) (74).

To further measure the potential cost of standard care/rehabilitation, the total number of physio- and occupational therapy treatments will be measured and compared between groups and centers.

### Sample Size

A 10% increase in muscle strength due to physical exercise is regarded as a clinically relevant change (62). Based on a mean 41.4 ± 7.6 (lower body muscle strength, kg ± SEM) (44) and a 10% increase, an alpha level of 0.05, and power of 80%, 106 children are needed. We expect that approximately 60 children with cancer aged 6–17 will be diagnosed per year at Copenhagen University Hospital, Rigshospitalet, Aarhus University Hospital, and Odense University Hospital. Assuming a 20% dropout rate, 2.2 years will be required to include the needed number of children with cancer (*n* = 127).

### Statistical Considerations

Constrained longitudinal data analysis is applied to evaluate the intervention effectiveness by using constrained (generalized) linear mixed models in two scenarios. In the first one, predictors will include follow-up time points categorized as 3 and 6 months to account for any non-linear effect and dummy variables representing the intervention group at 3- and 6-month follow-ups, respectively. In the second scenario, the time variable will be treated as a continuous variable and an interaction between treatments (binary-coded, 1 representing intervention group), and the time variable will be included instead. Normal distribution will be applied on continuous outcome muscle strength, while binomial distribution will be applied on binary outcome metabolic syndrome. Baseline characteristics, such as age (as a continuous variable), gender, and type of cancer (categorized as solid, CNS, and hematologic tumors), will be included additionally as covariates in both scenarios. Patient identity will serve as a random intercept. Likelihood ratio tests based on maximal likelihood will be applied for the model selection of the fixed effects to determine linear or non-linear associations. Benjamin-Hochberg procedure will be applied to reduce the false discovery rate due to multiple comparisons. The level of significance is 0.05.

### Data Management

Questionnaire data will be directly uploaded and stored on a secured server for sensitive data (REDCap). All other assessed data will be uploaded to the same server by all collaborators.

General Data Protection Regulations (GDPR EU) will comply with national and international law.

A data processing agreement with all collaborators will be made before any samples are shared for analysis.

## Discussion

This national randomized controlled study has the potential to investigate the short- and long-term effects of structured exercise in children and adolescents during anti-cancer treatment with a follow-up time into survivorship, 1 year after ended cancer treatment.

This study is based on almost a decade of experience within the research group conducting physical activity interventions for children with cancer through the RESPECT project. This experience has been extensively incorporated in this study; the chosen intervention, design, and choice of comparators.

### Intervention

The INTERACT study will use an exercise training intervention that may be complex due to the integrative design with individually targeted exercise prescriptions, i.e., we will not be able to present a generic exercise program that can accommodate all age groups, diagnosis, and logistical challenges. However, it does provide general guidelines for training modifications, exercise intensities, training accumulation, and suggestions for adequate rest and recovery during the first 6 months of cancer treatment. This will further provide a template for long-term exercise programming and long-term physical conservation (or even improvements) after ended cancer treatment. It will further provide evidence of the necessity of long-term exercise programming, appropriate testing, and monitoring to provide adequate physical exercise intervention, preserving strength and physical function during treatment. This will prepare children and adolescents for a normalized lifetime of exercise and active leisure activity after ended cancer treatment.

To maintain adherence and motivation throughout a 6-month training intervention, we expect weekly supervision of the training to be necessary. We therefore expect that exercise interventions with weekly supervision will have higher adherence rates, since participants will be more motivated, resulting in increased effects on muscle strength, markers of dysmetabolism, physical function, and levels of physical activity during and after treatment compared to unsupervised home-based training (active controls).

### Design

We have chosen a randomized controlled design to provide evidence of the potential effectiveness of integrative neuromuscular training in children and adolescents during cancer treatment. This will allow us to minimize confounding factors, such as geographical differences in patient uptake and usual care at each center, which was considered a limitation to the previous RESPECT study (18).

This study includes all malignant diagnoses of pediatric cancer. Due to different treatment protocols, length of hospitalization, and the potential dysfunctions and side effects from the cancer disease itself, this creates heterogeneity within and between groups. We choose to include all diagnoses, firstly to secure sufficient power in the study population within a reasonable timeframe, thereby minimizing bias due to changes and development of treatment protocols. Secondly, and most importantly, by including all cancer diagnoses, which we have shown are both motivated and trainable (13, 18), we will increase the generalizability and external validity of this study. To minimize heterogeneity, the groups will be stratified by sex, pubertal stage, and diagnosis.

### Choice of Comparators

A potential pitfall within the INTERACT study design may be the choice of using an active control group and performing a home-based intervention instead of using a passive comparator (i.e., usual care). Experiences from the two centers used as passive comparators within the RESPECT project showed that children or their parents are more likely to decline participation (up to 46%) or not adhere to scheduled assessment if placed in the passive control group (18). Furthermore, we found it ethically obligatory to be able to inform the participants and parents in the active control group, considering that they had accepted participation in an exercise intervention, about the potential benefits of physical activity and exercise during treatment, and to provide them with examples of body-weighted exercises.

The current evidence substantiates our hypothesis that adherence, and thereby potential effects, in a supervised exercise intervention will be higher than in home-based interventions.

The current intervention studies in hospitalized children with cancer are based on either home-based or supervised exercise. Adherence rates in these two types of interventions differ substantially from one another; home-based and supervised interventions report a weighted mean adherence of 64.3% (range 37–80) (35, 51, 52, 77) and 88.6% (range 85–100) (31, 32, 48, 49, 78), respectively. Logically, the studies with low adherence to exercise report either no effect or a small, non-significant effect on physical function or fitness, compared to usual care in current studies using home-based interventions. To be effective, physical intervention studies should therefore require a minimum degree of supervision and that non-supervised, home-based interventions correspond to usual care.

Accordingly, we believe that our active control group has close similarities to an adequate group receiving usual care. We also believe that this study will be able to demonstrate that information on physical exercise alone cannot be regarded as a sufficient alternative to supervised physical exercise.

In conclusion, physical activity and exercise interventions are regarded as a safe and feasible method to counteract treatment and inactivity-related side-effects in children and adolescents with cancer; nevertheless, large-scale studies are needed to draw definite conclusions regarding the effectiveness of physical exercise interventions (12, 18). An age-appropriate integrative exercise intervention started immediately after treatment initiation is a promising strategy to reduce the anti-cancer treatment-related side effects.

This research project can potentially change the pediatric exercise oncology and rehabilitation field. The project strives to document between-group changes in strength and physical function, thereby advancing from concluding safety and feasibility measures to report not only a preservation of physical function but significant improvements in children and adolescents' physical function after 24 weeks of treatment compared to treatment initiation. We will achieve our results using a pragmatic, low-cost, and time-efficient training intervention that is appropriately developed for both children and adolescents and can be adapted to both hospital and home settings. This intervention can therefore relatively easily be implemented into current clinical practice.

### Ethics and Dissemination

The study will comply with the Helsinki II Declaration. The study has been peer-reviewed and approved by the Danish National Committee on Health Research Ethics (Approval Number: H-20040897), and data handling is approved by the Danish Data Protection Agency (jr. nr.: P-2021-14).

### Consent to Participate

Written informed consent will be obtained before inclusion to the study by a member of the research staff (project nurse or physiotherapist) alongside information about the potential risks and benefits of participating in the study. This includes information concerning the child or adolescent's privacy rights and the investigator's disclosure obligations.

Adolescents (aged 15–17.9 years) will receive oral and written information specifically adapted to this age group. If a patient does not wish to participate, this is respected regardless of the parent's acceptance.

### Risks and Adverse Reactions

The project is expected to cause limited risks, side effects and discomfort.

Integrative neuromuscular training and isometric muscle strength tests are associated with exertion and shortness of breath and may in some cases feel strenuous. If either the intervention staff or the participant-assigned physician assess that participation is unsafe, the training session or test will be canceled. Reasons for canceling an intervention training session or test include thrombocyte counts <10 billion/l, hemoglobin <5 mmol/l or systolic blood pressure <95 mm Hg.

### Dissemination Policy

The results of this study will be presented in scientific peer-reviewed journals and at international conferences. Authorship eligibility follows the Vancouver Recommendation.

## Data Availability Statement

The datasets generated and/or analyzed during the current study are not publicly available due Danish and EU personal data legislation but are available from the corresponding author on reasonable request.

## Ethics Statement

The studies involving human participants were reviewed and approved by Danish National Committee on Health Research Ethics. Written informed consent to participate in this study was provided by the participants' legal guardian/next of kin.

## Author Contributions

This protocol article was primarily drafted by HL, KM, PS-A, MF, and JC. HZ drafted the statistical considerations paragraph and is responsible for the statistical analysis. All authors (PS-A, MF, KM, AP, LH, AF, KS, HH, SL, HZ, JC, and HL) have substantially contributed to the study design and conception of the intervention and will be involved in data collection, analysis, and/or manuscript preparation as the study proceeds. All authors have revised and approved the final manuscript.

## Funding

The INTERACT study has been peer-reviewed and funded by the Danish Childhood Cancer Foundation (Grant numbers: 2019-5954 and 2020-6769), the Research Fund of Copenhagen University Hospital (Grant number: E-22597-01), Capital Region of Denmark's Research Foundation for Health Research 2020 (Grant number: A-6868), Helsefonden (Grant number: 20-B-0409), Danish Cancer Research Fund (Grant number: FID2157728), the Research Fund of the Association of Danish Physiotherapists (Grant number: R23-A640-B408), and Fabrikant Einar Willumsen's Memorial Scholarship (Grant number: N/A). This work is part of Childhood Oncology Network Targeting Research, Organization & Life expectancy (CONTROL). None of the funders will have any role in the design of this study, in the collection of data, the analysis, the interpretation of data, or the dissemination of data.

## Conflict of Interest

The authors declare that the research was conducted in the absence of any commercial or financial relationships that could be construed as a potential conflict of interest.

## Publisher's Note

All claims expressed in this article are solely those of the authors and do not necessarily represent those of their affiliated organizations, or those of the publisher, the editors and the reviewers. Any product that may be evaluated in this article, or claim that may be made by its manufacturer, is not guaranteed or endorsed by the publisher.

## Abbreviations

CNS: Central Nervous System
DXA (scan): Whole-Body Dual-Energy X-Ray Absorptiometry
HDL: High-Density Lipoprotein (cholesterol)
ICF: international classification of functioning, disability, and health
INT: Integrative Neuromuscular Training
INTERACT (study): Integrative neuromuscular training in adolescents and children treated for Cancer
OR: OddsRatio
RESPECT (project): REhabilitation including Social and Physical activity and Education in Children and Teenagers with cancer.
