# Supplementary material for: Integrative Neuromuscular Training in Adolescents and Children Treated for Cancer (INTERACT): Study Protocol for a Multicenter, Two-Arm Parallel-Group Randomized Controlled Superiority Trial
Source: Front Pediatr. 2022 Mar 14;10:833850. doi: 10.3389/fped.2022.833850 (PMC8964065; doi:10.3389/fped.2022.833850)
Supplement: Supplementary file 1 [file Data_Sheet_1.PDF]

Supplementary figure 1: Home-based training program

Description of exercises (Active control group)

LOWER BODY EXERCISES:
Calf Raises (easy):
With a hip-width distance between the feet, stand on a mat or the floor. Lift your heels so that you are now standing on your toes. Lower yourself to the starting position and repeat.

Squat (intermediate/hard):
With a shoulder-width distance between the feet, bend down to an approx. 90-degree angle at the knees, then return to the starting position. Keep your back straight and look ahead.

Hip Thrust (intermediate):
Lie on your back, bend your legs and let your feet rest on the mattress/bed. Brace your stomach and buttocks. Lift your buttocks from the mat until there is a straight line between the shoulder and the knee.

UPPER BODY EXERCISES:
Pushups Against Wall (easy):
(As below, leaning against wall)

Pushups, Resting on Knees (intermediate):
Place the knees on the floor, keep your upper body fixed with outstretched arms and a shoulder-wide grip. Lower your body to the floor and push back.

Pushups (hard):
Stand on your toes, keep your upper body fixed with outstretched arms and a shoulder-wide grip. Lower the body to the floor and push back.

STRETCHING EXERCISES:
Calf stretch:
Support yourself against a wall and place one sole of the foot up against the wall, lowering the heel on the floor. Keep the knee fully extended/stretched. Gently press the hip forward until feeling a stretch on the back of the lower leg. Hold for 30 sec. and change leg.

Front thigh stretches:
Sit on the floor with legs outstretched. Try to lean your body forward and touch your toes. Hold for 30 sec.

Supplementary figure 1: Description and illustration of exercises in the active control group.
