# Supplementary material for: Integrative Neuromuscular Training in Adolescents and Children Treated for Cancer (INTERACT): Study Protocol for a Multicenter, Two-Arm Parallel-Group Randomized Controlled Superiority Trial
Source: Front Pediatr. 2022 Mar 14;10:833850. doi: 10.3389/fped.2022.833850 (PMC8964065; doi:10.3389/fped.2022.833850)
Supplement: Supplementary file 2 [file Data_Sheet_2.PDF]

Supplementary figure 2: Treatment adjusted training plan, example

6 months of INT training, Acute Lymphocitic Leukemia, LOW-RISK																								
Month	1				2				3				4				5				6			
Week	1	2	3	4	5	6	7	8	9	10	11	12	13	14	15	16	17	18	19	20	21	22	23	24
Chemo (cycle)	x	x	x	x	x	x		x				x			x			x	x	x				
Radiation																								
Surgery																								
Mesocycle (theme)	Adaption		Individualisation		Progressive overload 1				Progressive overload 2				Progressive overload 3				Progressive overload 4				Transition			
Contents	Warm up Introduction to equipment Matching of expectations		Warm Up (individualized) Motor Skills Strength exercises (BW) Incorporate own exercises		Motor Skill and Strength/Power				Strength/Power + Motor Skill				Strength/Power +Motor Skill +Endurance				Strength/Power +Motor Skill +Endurance +Agility				Strength/Power +Motor Skill +Endurance +Agility			
Training sessions/week	2	2	2	2	2	2	2	3	3	3	3	3	3	3	3	3	3	3	3	3	3	3	3	3
Number of primary exercises	1 to 2				1 to 2				≥2				≥2				≥2				≥2			
Total number of exercises	1 to 3				1 to 3				2 to 4				3 to 5				3 to 5				4 to 6			
Time per training session	15	15	20	20	15	15	15	20	15	20	30	35	20	20	30	20	25	30	35	35	20	25	30	35
Pause between exercises	2 min →																							30 sec
Workload (1-10)	2	3	4	3	2	1	1	3	2	2	3	4	2	3	5	2	4	6	7	8	2	4	6	8

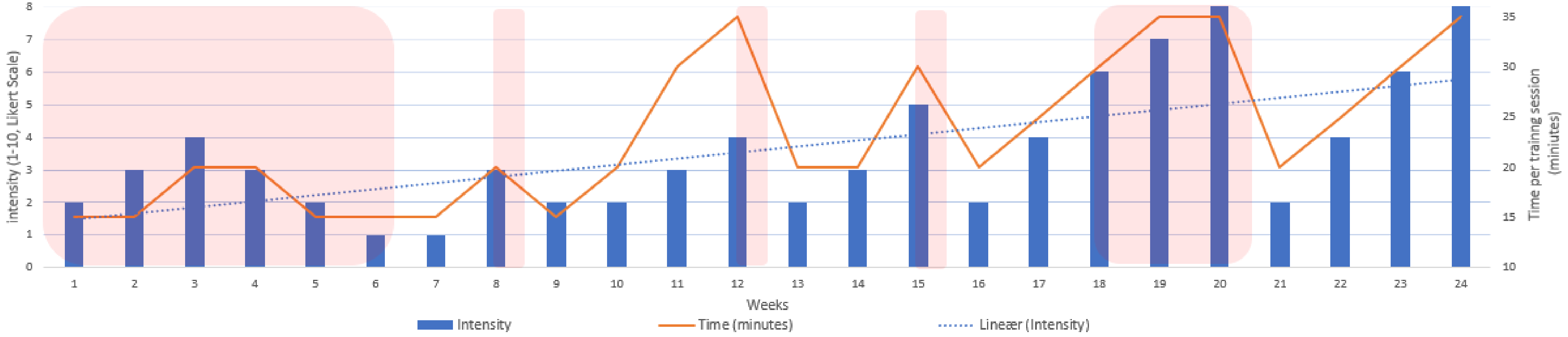

Legend: 24-week example of a tentative individual training plan adjusted to a low-risk treatment protocol for acute lymphoblastic leukemia (ALLtogether 2018; ClinicalTrials.gov identifier: NCT04307576). The bottom diagram shows the inverse relationship between training intensity (blue pillars) and time per training session, including resting periods, (orange curve), according to chemotherapy treatment cycle. The dotted curve shows the linear progression in intensity throughout six months of intervention. The primary purpose in alternating the intensity and time per training session, is to accommodate the variation of treatment-related side effects. Therefore, the intensity of training will be low, and the total training time will be longer, when the burden of side effects will be high and vice versa.
